# Effect of P content on microstructure and wear properties of ZCuPb_20_Sn_5_ alloy

**DOI:** 10.1039/c9ra04651h

**Published:** 2019-10-29

**Authors:** Xin Zhang, Xiao-yan Ren, Xu Hong, Xiang-yu Gao

**Affiliations:** Technology Center/General Institute of Emergency Research, Xinxing Cathay International Group Beijing 100070 China kmzx201@163.com; School of Materials Science and Engineering, North University of China Taiyuan 030051 China xh725@263.net; Department of Automotive Engineering, Tsinghua University Beijing 100083 China

## Abstract

The properties and friction behavior of ZCuPb_20_Sn_5_ modified with P were investigated. With the P addition and content increase, the second phase appeared and gradually increased in amount. Also, the microstructure of ZCuPb_20_Sn_5_ was refined and evenly distributed. The addition of P had a beneficial effect on the microstructure and properties of ZCuPb_20_Sn_5_. As the P content increased, the hardness and tensile strength of ZCuPb_20_Sn_5_ increased, but the elongation, the friction coefficient and the wear rate decreased. The wear mechanism of ZCuPb_20_Sn_5_ was mainly adhesive wear, and a small amount of debris was produced. As the P content increased, the anti-wear resistance of ZCuPb_20_Sn_5_ deteriorated.

## Abstract 2

The properties and friction behavior of ZCuPb_20_Sn_5_ modified with P were investigated.

## Graphical abstract

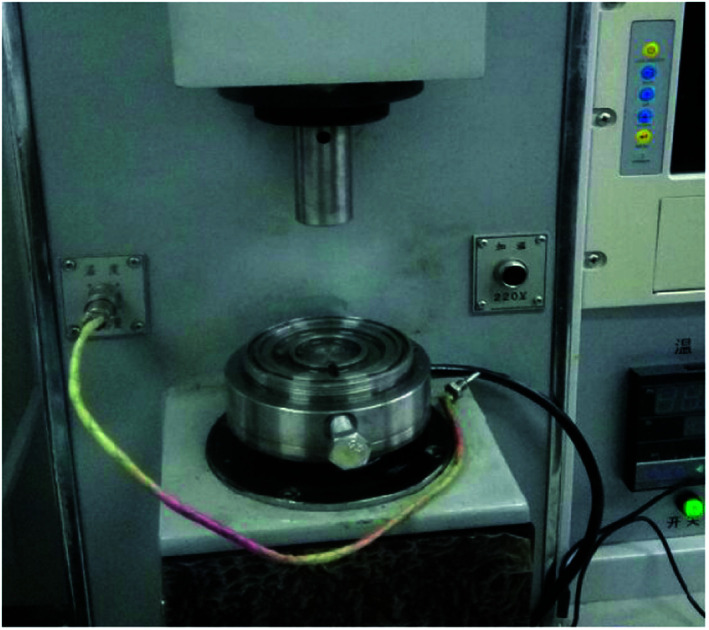

## 1 Introduction

Cu alloys are widely utilized, due to their good casting performance, wear resistance, corrosion resistance and mechanical properties. Cu alloys are often used in the manufacturing of various machines to sustain heavy loads and high-speed operation of shaft sliding bearings, such as sleeves and bush bearings as well as in automobiles, ships, metallurgy, machinery and other industrial fields. With the rapid scientific and technological progress as well as economic development, the high requirements for Cu alloys increased rapidly.^1^ High Pb–tin bronze alloys, which are currently widely used in engine bearings with high-speed and heavy load, have excellent dry friction resistance, high thermal conductivity and fatigue resistance.^2,3^ Currently, one of the main ways to improve the performance of tin–bronze alloys is to add alloying elements, such as rare earths, Ni, Pb, Fe, Mn, Al and P.^4,5^ M. Aksoy^6^ and H. Turhan^7^ used the matrix alloying method to add Fe, Mn, Si, P, S and other elements to tin–bronze alloys, in which, the hard Mn_5_Si_3_ and Fe_3_S dispersed phases precipitated from the matrix, improving the sintering and abrasion resistance. Qi Zhan Jun^8^ studied the microstructure of high lead bronze alloy with different amounts of lead, S and rare earths, which demonstrated that the lack of S addition led to the severe lead segregation, occurring in the centrifugal casting of high lead bronze alloys. Also, the lead segregation was in the form of lumps and ribbons. The lead segregation was effectively controlled when S was added. With the addition of rare earths, the strip or block lead in the alloy changed into point-like or spherical lead. In this case, the point-like or spherical lead was uniformly distributed, while the degree of lead segregation decreased as the rare earth amount increased. C Nobel^9^ improved the tensile strength and wear resistance of lead–tin Bronze through the addition of traces of rare earths and Pb, which refined the microstructure and improved the mechanical properties. The tensile strength of the Pb–Sn bronze alloy reached 192 MPa when the pouring temperature, the contents of lanthanum–cerium-rare earth and P were 1250 °C, 0.2% and 0.5%, respectively. In the study of the effect of Fe traces on the microstructure and properties of semi-continuous casting of tin bronze, X. Y. Mao^10^ demonstrated that the addition of Fe played an apparent role in grain refinement, reducing the columnar crystal sizes, along with the microstructure stress caused by the columnar crystals, while improving the subsequent processing performance.

P plays a specific role in high temperature alloys and is generally regarded as a harmful non-metallic impurity.^11,12^ However, few studies and reports on the role and mechanism of P in high temperature alloys have been reported. In recent years, significant attention has been paid to the role of P in alloys both domestically and abroad. A. M. Dubey^13^ and P. Sahlot *et al.*^14^ studied the effect of P on the properties and segregation of superalloys. It was concluded that P, within a certain content range, could also significantly improve the life of alloys. Moreover, and the addition of an appropriate amount of P could improve the creep and rupture properties of alloys. This occurred because the solidification segregation of deformed superalloys was basically eliminated or highly reduced subsequently to hot working and heat treatment. Simultaneously, the P atoms were segregated at the grain boundary, changing the bonding relationship among main elements on the grain boundary, forming certain large clusters. In addition, the bonding force among atoms increased, improving the grain boundary strength, while changing the precipitate morphology at the grain boundary. This led to the durability and creep properties improvement of the alloy.^15^

At present, the applications of P in lead–tin bronze alloys were seldom studied. Consequently, the reasonable amount control of P in the alloy has become a difficult problem. In this paper, the effects of P on the structure and properties of lead–tin bronze alloy were mainly analyzed on the basis of existing research results. This was conducted, in order to comprehensively understand the effect, function and mechanism of P on lead–tin bronze ZCuPb_20_Sn_5_, as well as to clarify the depth or process of the current research along with the existing problems, to design more targeted future research.

## 2 Materials and methods

### 2.1 Experimental

The raw materials were electrolytic copper, lead ingot, tin ingot, zinc ingot, pure nickel and P–copper alloy with a P content of 13.5%. The purity of all raw materials was 99.99%. The smelting well type resistance furnace was a SG2-12–13, the rated voltage was 380 V and the rated temperature was 1300 °C. P was twice added as a deoxidizer. When the copper block was completely melted, 1/2–2/3 of P copper were added for initial deoxidization, whereas the remaining P–copper was added subsequently to all other alloying element additions. The alloying elements of Ni, Zn, P and Sn were added in sequence, according to the melting point values of the elements from high to low. When the melt temperature was 1200 °C, the fully stirred melt was quickly cast into a high temperature preheated metal mold. When the casting temperature of alloy melt was too low, the fluidity and mold filling integrity were affected. This was not conducive to produce uniform composition and casting. When the pouring temperature of alloy melt was too high, the alloy lost certain amounts of alloying elements while producing fumes, which severely affected both microstructure and properties. Therefore, the pouring temperature deviation of alloy melt did not exceed 20 °C. When the molten alloy was poured, the flow rate was high, the liquid column was short and the protective atmosphere prevented oxidation. Each process must be strictly controlled to avoid inhalation of gases and significant loss of material. Subsequently to casting for 5 min, the mold was opened and the casting parts were removed. Following pouring, the casting parts were processed to produce tensile, metallographic and friction pin specimens.

**Fig. 1:**
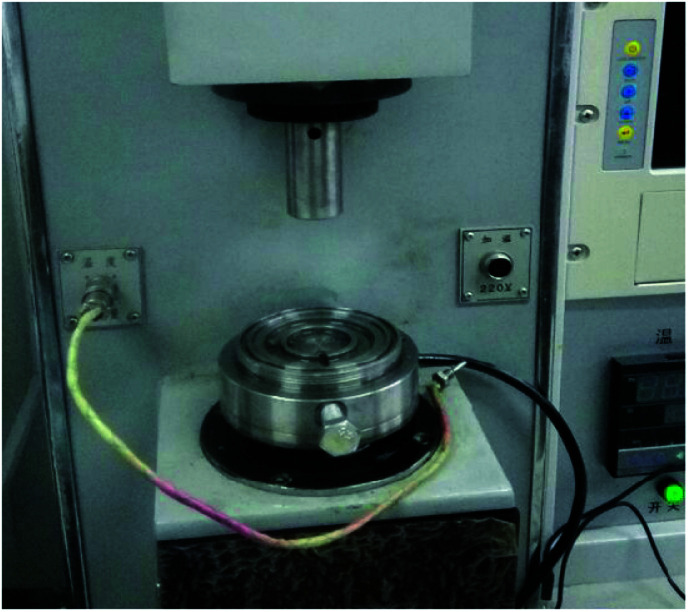
MMW-1A friction and wear tester.

**Fig. 2:**
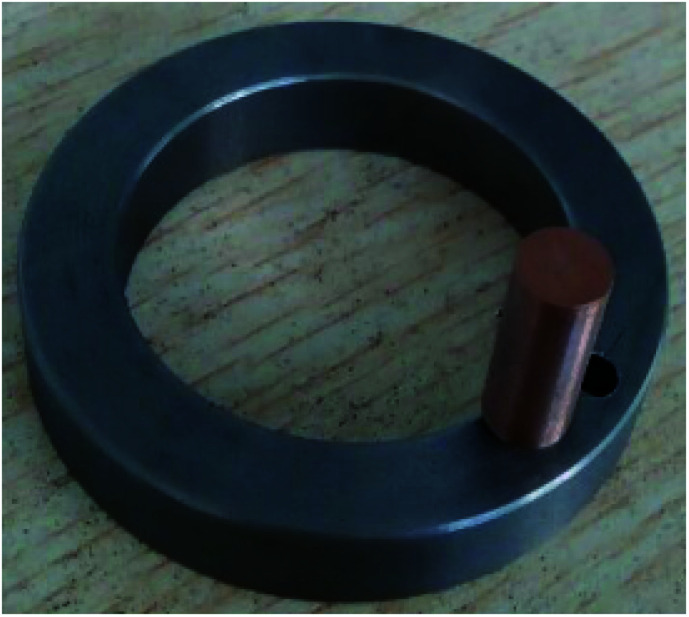
Friction pair.

The friction and wear tests were carried out with a MMW-1A friction and wear tester, as presented in Fig. 1. The friction pair in the friction experiment is presented in Fig. 2. The friction pair must satisfy the following conditions: hardness exceeding 280 HB and the surface roughness below 0.8. The friction pin was selected from the lead tin bronze samples from the above experiment. The samples were polished, polished and cleaned. The wear amount was measured with an electronic balance of 1/10 000 precision. The relationship between wear and abrasion was obtained through the wear volume calculation. The P friction influence analysis on the wear properties of tin bronze alloy was mainly based on the friction coefficient and wear volume.

The corrosion products were investigated through grazing incidence X-ray diffraction with Cu K_α1_ (*λ* = 0.154056 nm) radiation at a constant incidence angle of 1° to the specimen surface. The crystalline phases were identified with JCPDS database cards.

### 2.2 Experimental procedure

According to the composition requirement of the ZCuPb_20_Sn_5_ alloy, 2% of nickel and different contents of P were added. The specific experimental scheme is presented in Table 1.

**Table 1:** Alloy experimental scheme

NO.	Cu (wt%)	Pb (wt%)	Sn (wt%)	Zn (wt%)	Ni (wt%)	P (wt%)
1	Remaining	20.04	5	1.75	2	0
2	Remaining	20.04	5	1.75	2	0.05
3	Remaining	20.04	5	1.75	2	0.1
4	Remaining	20.04	5	1.75	2	0.2
5	Remaining	20.04	5	1.75	2	0.3

## 3 Results and discussion

### 3.1 Microstructure analysis

#### (1) Metallographic analysis

**Fig. 3:**
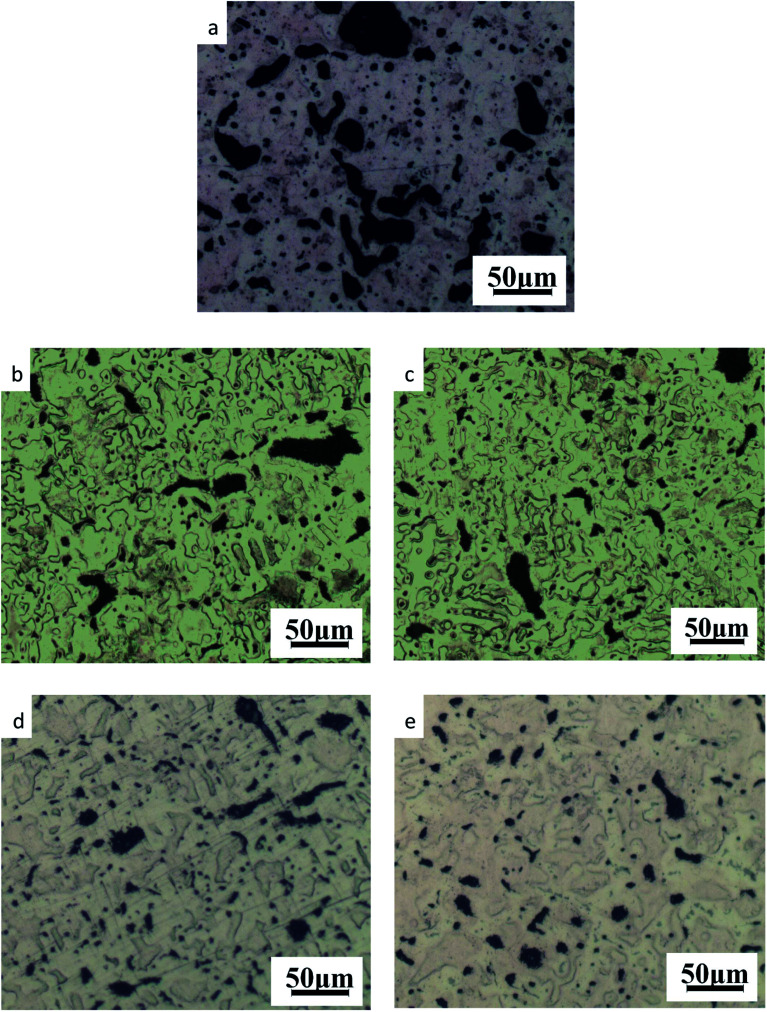
Microstructure of ZCuPb_20_Sn_5_ with different content of P: (a) P = 0.0 wt%; (b) P = 0.05 wt%; (c) P = 0.1 wt%; (d) P = 0.2 wt%; (e) P = 0.3 wt%.

The microstructures of the ZCuPb_20_Sn_5_ alloys with different contents of P (0%, 0.05%, 0.1%, 0.2% and 0.3%) are presented in Fig. 3 (a), (b), (c), (d) and (e), respectively. The gray-white matrices were the α solid solution with copper as the matrix, while the gray dendritic massive structure was a tin-rich solid solution.

As it could be observed from Fig. 3, without the addition of P, a high amount of lead was gathered at the microscopic pores, to form large particles before it could be dispersed. This led to the uneven distribution and high segregation of lead.

When the P–Cu alloy containing 0.05% of P was added, the large lead particles decreased in size, while the large spherical particles decomposed into small worm-like particles. The gray massive α + δ eutectoid was distributed irregularly and inhomogeneously.

When the P–Cu alloy containing 0.1% of P was added, the large lead particles apparently decreased in size and continued to transform from large spherical into worm-like. One portion of the gray bulk α + δ eutectoid was irregularly and unevenly distributed, whereas the other part was evenly distributed among dendrites.

When the P–Cu alloy containing 0.2% of P was added, the spherical particles had basically transformed into vermicular particles. The volume of gray bulk α + δ eutectoid increased and distributed among the dendrites. Also, the dendrites became coarse.

When the P–Cu alloy containing 0.3% of P was added, the Pb particles changed from wormlike to relatively small spherical particles and the gray massive (α + δ) eutectoid was distributed in the tissues producing chrysanthemum-like uniformity and irregularity. Moreover, the segregation of Pb particles was not apparent.

From the aforementioned microstructure, it could be concluded that the addition of P reduced the segregation of lead, leading to a homogeneous matrix microstructure. However, when the content of P and copper exceeded 0.1%, the volume of gray bulk (α + δ) eutectoid gradually increased, while the dendrites gradually became coarse. As the solubility of P in solid solution was 0.1, the eutectic structure (α + Cu_3_P) could be formed when the content of P exceeded 0.1%. The Cu_3_P phase was hard and brittle, often composed of binary and ternary eutectic phases with α and δ phases.

#### (2) SEM and EDS analysis

**Fig. 4:**
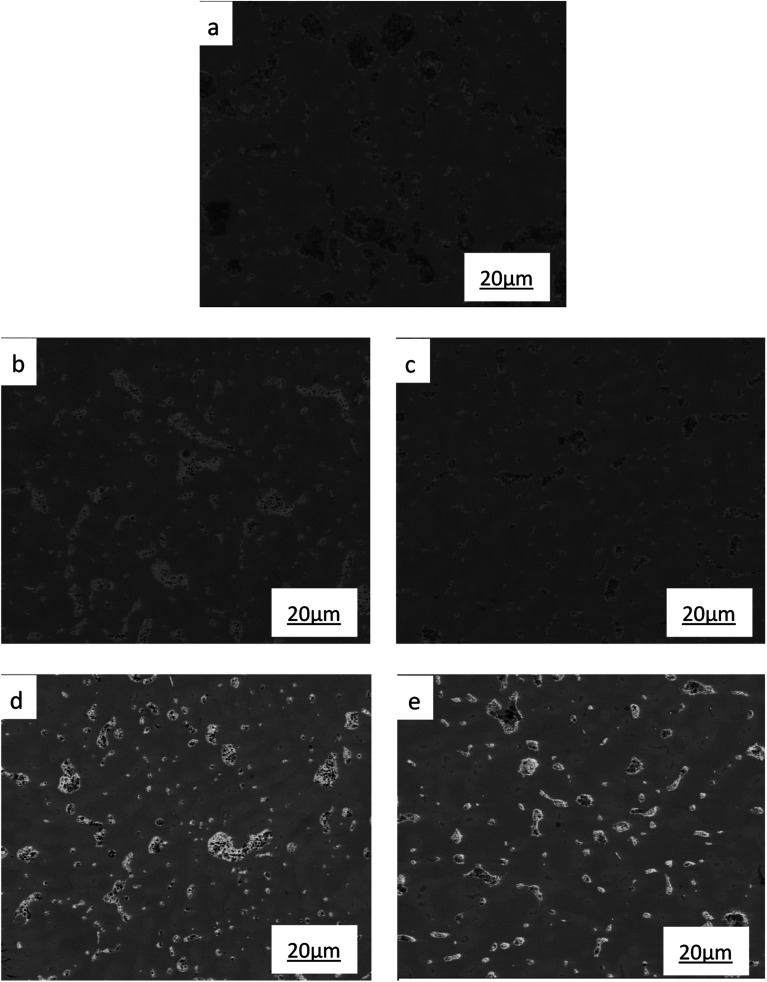
SEM images of ZCuPb_20_Sn_5_ with different content of P: (a) P = 0.0 wt%, (b) P = 0.05 wt%, (c) P = 0.1 wt%, (d) P = 0.2 wt%, (e) P = 0.3 wt%.

The SEM microstructures of the ZCuPb_20_Sn_5_ alloy with different P contents (0%, 0.05%, 0.1%, 0.2% and 0.3%) are presented in Fig. 4 (a), (b), (c), (d) and (e), respectively. It could be clearly observed that the change pattern of lead particles was consistent with Fig. 3. As the P content increased, the large lead particles became wormlike and consequently decomposed into spherical particles, evenly distributed in the tissue, reducing the segregation of lead. Simultaneously, a black gray creeping Cu_3_P phase appeared due to P addition. At 500 times magnification of the scanning state, when the content of P was 0.3%, the Cu_3_P phase with fine black-gray vermicular strips was barely visible.

**Fig. 5:**
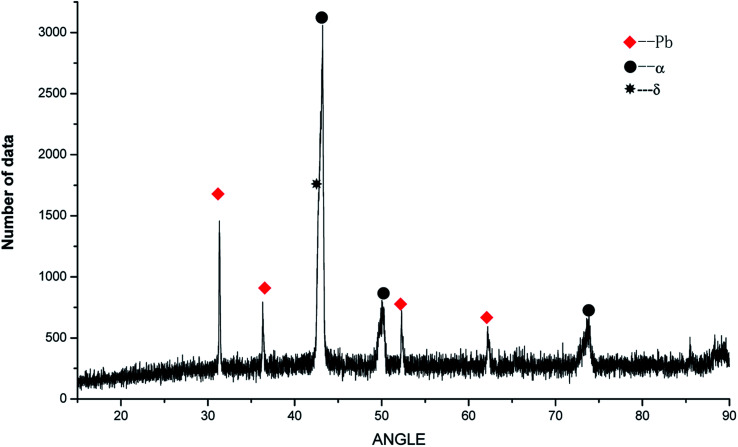
XRD analysis of ZCuPb_20_Sn_5_ alloy with different P.

To determine the effects of P on the alloy phase type and chemical composition of the second phases, the XRD analysis was used and is presented in Fig. 5. It could be observed that the main phase composition of the alloy consisted of Pb + α +δ phases and it was found that the major secondary phase in the alloy was Cu_3_P.

**Fig. 6:**
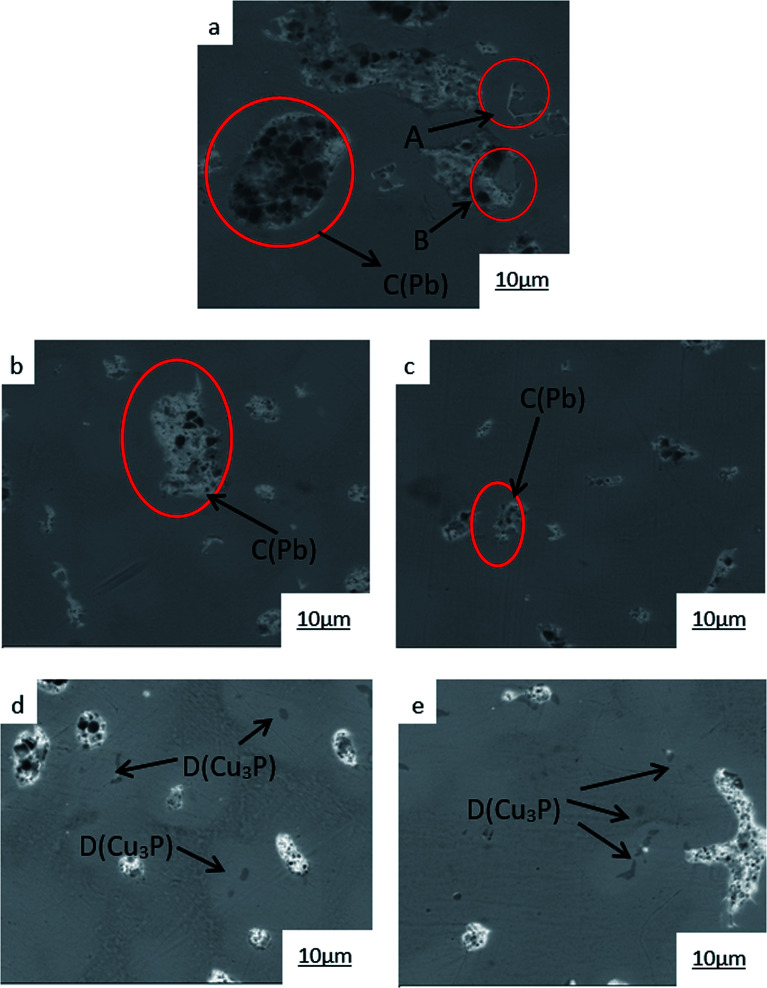
SEM images of ZCuPb_20_Sn_5_ with different content of P: P = 0.0 wt% (a), P = 0.05 wt% (b), P = 0.1 wt% (c), P = 0.2 wt% (d), P = 0.3 wt% (e).

**Fig. 7:**
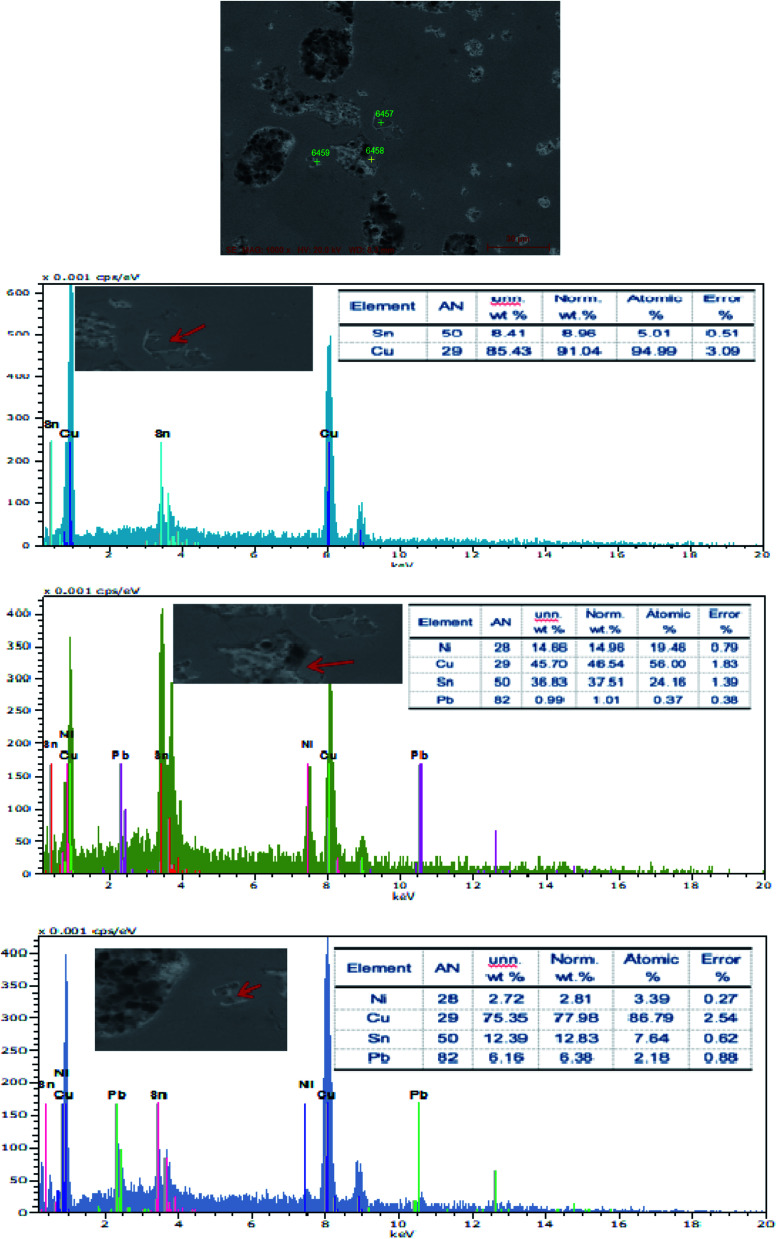
EDS spectra of ZCuPb_20_Sn_5_ without P.

**Fig. 8:**
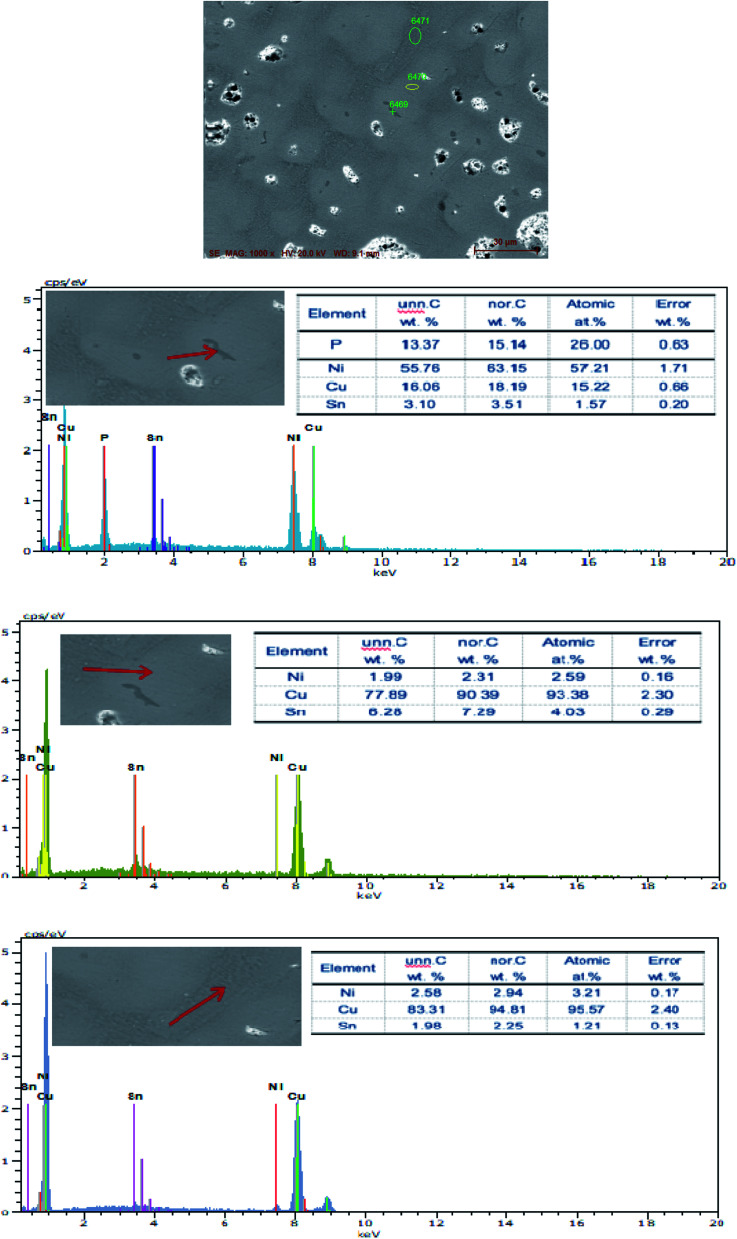
EDS spectra of ZCuPb_20_Sn_5_ modified by 0.5% P.

The SEM figures of ZCuPb_20_Sn_5_ alloy with different P contents (0%, 0.05%, 0.1%, 0.2% and 0.3%) at 1000 times are presented in Fig. 6 (a), (b), (c), (d) and (e), respectively. EDS spectra analysis of ZCuPb_20_Sn_5_ alloy prior to and following modification with phosphorus are presented in Fig. 7 and 8, respectively. As it could be observed in the SEM photographs and EDS spectra, the microstructure of ZCuPb_20_Sn_5_ alloy without P–Cu alloy addition had the large aggregates of black and gray granular lead. Combined with the energy spectrum, it could be observed that the gray massive microstructure A was mainly the δ phase. This was the solid solution based on the electronic compound of Cu_31_Sn_8_. Similarly, the bulk microstructure B was also the tin-rich region, with high tin content and significant nickel aggregation, indicating that the microstructure was extremely inhomogeneous. The fine black particles were the lead particles. The large black massive particles were the grown lead particles, which were embedded in microscopic pores. When the P–Cu alloy containing 0.05% P was added, certain Cu_3_P compounds could be faintly observed. Also, the large bulk lead particles decreased in amount and the bulk δ matrix could not be observed. When the P content was 0.1%, the small wormlike Cu_3_P compound was observed, the large lead particles disappeared and the microstructure was relatively fine and uniform. When the P content was 0.2%, the Cu_3_P compound increased in size, and the lead particles were small spheres without large aggregates. The gray-white region was the (α + δ) phase with copper and tin as the matrix. As the P content increased to 0.3%, Cu_3_P compounds could be observed and the shape of Cu_3_P compounds had changed.

From the influence analysis of different P contents on the microstructure of the alloy, the Pb particle microstructure became even less homogeneous as the P content increased.

### 3.2 Effect of P modification on morphology and distribution of lead

**Fig. 9:**
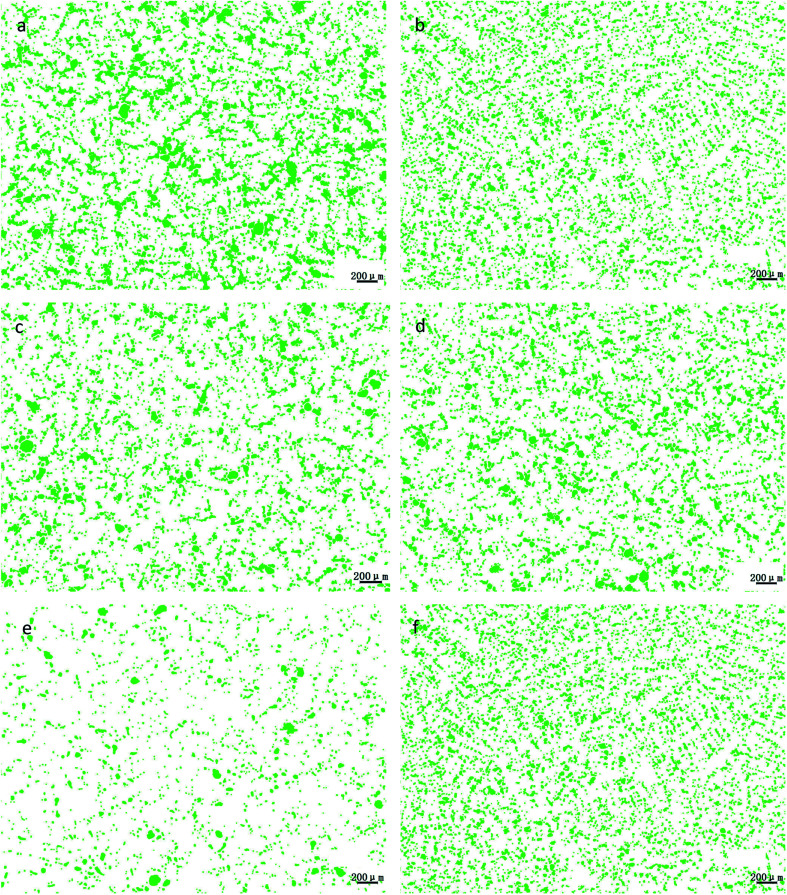
Morphological changes of lead particles in leaded tin bronze with different P contents: (a) P = 0.0 wt%; (b) P = 0.05 wt%; (c) P = 0.1 wt%; (d) P = 0.2 wt%; (e) P = 0.3 wt%; (f) P = 0.5 wt%.

**Fig. 10:**
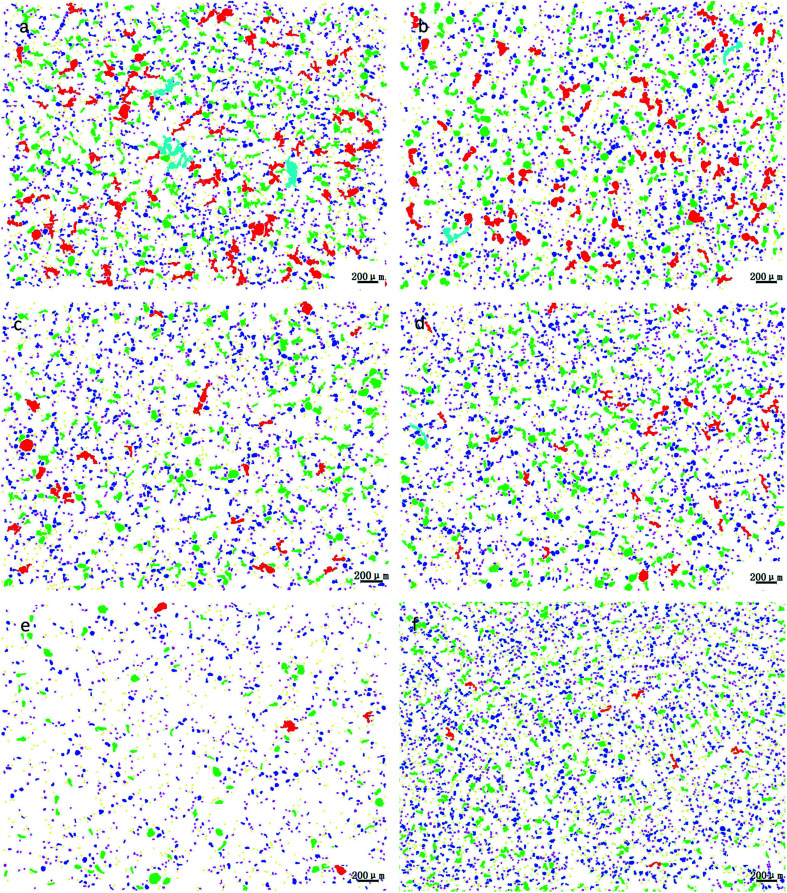
Changes of lead particle size distribution in lead tin bronze with different P contents: (a) P = 0.0 wt%; (b) P = 0.05 wt%; (c) P = 0.1 wt%; (d) P = 0.2 wt%; (e) P = 0.3 wt%; (f) P = 0.5 wt%.

Through the comprehensive analysis of the data in Fig. 9, 10 and Table 2, it could be observed that as the phosphorus content increases, the large lead particles gradually disappeared and transform into very small particles. Among them, the yellow particles, the purple-pink particles, the dark blue particles, the green particles, the red particles and the blue-green particles were of 0–15 μm, 15–30 μm, 30–60 μm, 60–120 μm, 120–250 μm and 250–500 μm in size, respectively. Only 4 particles existed in the 250–500 μm size range without phosphorus or copper. When the phosphorus addition was 0.05%, it was reduced to 1. Without phosphorus and copper, 84 particles were in the range of 120–250 μm and 29 particles were in the range of 0.05% of phosphorus. Following, as the phosphorus amount increased, the particle numbers in this range gradually decreased. When phosphorus content was 0.5%, only 7 lead particles existed in this range. From this data, it could be observed that the addition of phosphorus could refine the morphology of lead particles.

**Table 2:** Size and number distribution of lead particles in lead–tin bronze alloy

Diameter (μm)	P: 0%	P: 0.05%	P: 0.1%	P: 0.2%	P: 0.3%	P: 0.5%
0–15	917	823	500	871	902	871
15–30	987	1089	821	1013	1090	1013
30–60	563	685	452	650	650	650
60–120	292	253	121	220	220	220
120–250	84	29	6	29	19	29
250–500	4	1	0	0	0	0
>500	0	0	0	0	0	0

### 3.3 Mechanical properties analysis

**Fig. 11:**
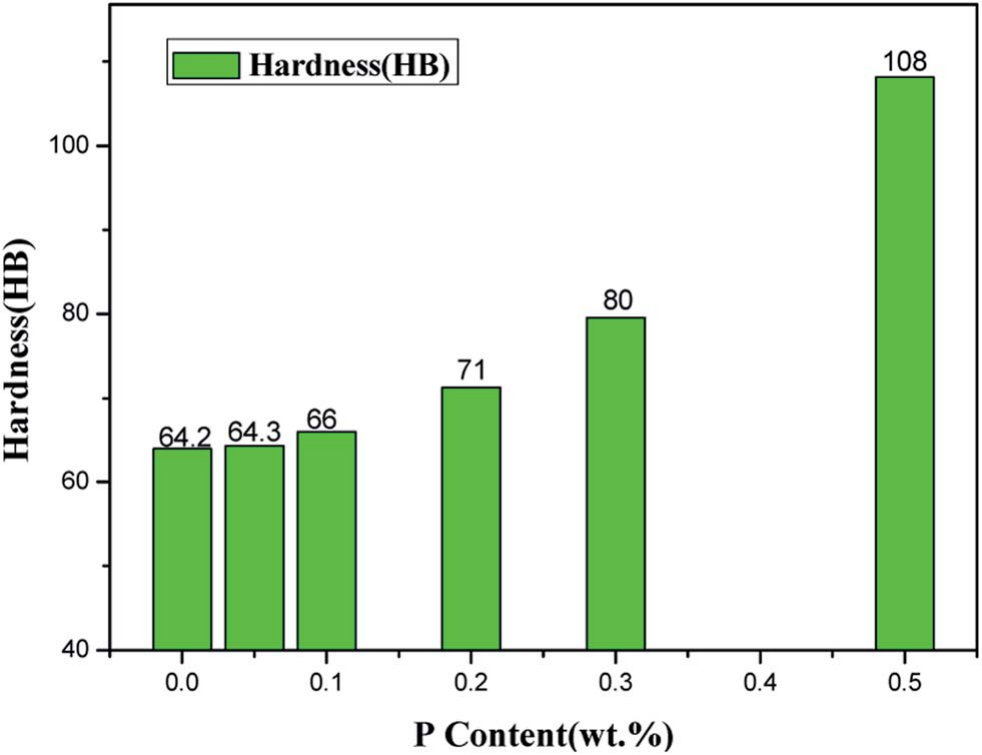
Hardness curve.

The hardness of ZCuPb_20_Sn_5_ alloy with different P contents was measured through experimentation. Each hardness value was measured five times and the average value was obtained. The Victorinox hardness curve of ZCuPb_20_Sn_5_ alloy with different P contents is presented in Fig. 11. As it could be observed from Fig. 11, when the P content was 0.05%, the hardness of ZCuPb_20_Sn_5_ alloy was basically unchanged from 55 HV to 57.02 HV. When the P content was 0.1%, the hardness of ZCuPb_20_Sn_5_ alloy was 58.78 HV. The hardness of ZCuPb_20_Sn_5_ alloy increased as the P content increased by 0.1%. When the P content was 0.2%, the hardness of ZCuPb_20_Sn_5_ alloy was 64.32 HV. When the P content was 0.3%, the hardness of ZCuPb_20_Sn_5_ alloy increased to 76.94 HV.

It was known that the addition of P improved the ZCuPb_20_Sn_5_ alloy hardness. The Cu_3_P compound was formed through the P–Cu alloy addition to the melt. The compound was hard and evenly distributed at the grain boundaries, which inhibited the segregation of lead. In this way, on the one hand, Cu_3_P compound inhibited the movement of dislocations, while as the number of Cu_3_P compound particles increased, the resistance to dislocation motion increased, resulting in the alloy hardness increase. On the other hand, the Cu_3_P particles were uniformly distributed at the grain boundaries, resulting in the formation of several crystalline cores during solidification, which could refine the grain and produce fine grain strengthening. For a certain range, as the P content increased, the effect of fine grain strengthening became more apparent, consequently enhancing the ability of the matrix to resist plastic deformation. This led to the increase of hardness and wear resistance of lead–tin bronze alloy. Therefore, as the P content increased, the hardness increased at the macro level.

When the content of tin and P reached to a certain content, the Cu_3_P and (α + δ) phases formed the ternary eutectic phases ((α + δ + Cu_3_P) phases), which deteriorated the properties of the alloy to a certain extent. Therefore, the content of P in the modified tin–bronze alloy should not exceed 0.5%, otherwise it would cause hot cracking during processing.

**Fig. 12:**
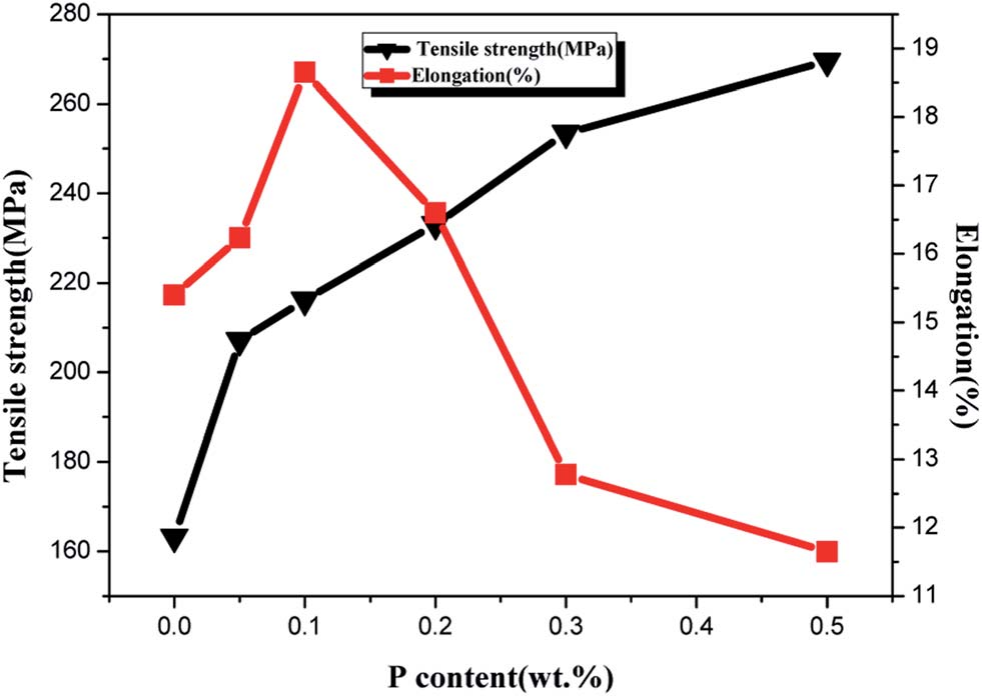
Elongation and tensile strength curve.

The elongation and tensile strength of ZCuPb_20_Sn_5_ alloy with different P contents were measured through experimentation, as presented in Fig. 12. As the P content increased, the tensile strength of ZCuPb_20_Sn_5_ alloy presented an increasing trend. The tensile strength of ZCuPb_20_Sn_5_ alloy was 187.65 MPa without the phosphor copper alloy addition. The tensile strength of ZCuPb_20_Sn_5_ alloy was 207 MPa when the mass fraction of P increased to 0.05%. Subsequently, the tensile strength increased linearly as the P content increased. When 0.3% of P was added, the tensile strength reached 248.49 MPa, which was increased by 61 MPa compared to the alloy without P–copper. The elongation of ZCuPb_20_Sn_5_ alloy increased first and consequently decreased as the P content increased. When the P content was 0.1%, the elongation of ZCuPb_20_Sn_5_ alloy reached the maximum of 17.8%. Following, the ZCuPb_20_Sn_5_ amount gradually decreased as the P content increased. When the P–Cu alloy with a mass fraction of 0.3% was added, the elongation of ZCuPb_20_Sn_5_ alloy decreased to 10%.

It could be preliminarily determined that as the P content increased, a high amount of Cu_3_P occurred, conmsequently improving the tensile strength of ZCuPb_20_Sn_5_ alloy. When the content of Cu_3_P continued to increase, the thermal brittleness increased and the elongation decreased.

### 3.4 Friction and wear performance analysis

**Fig. 13:**
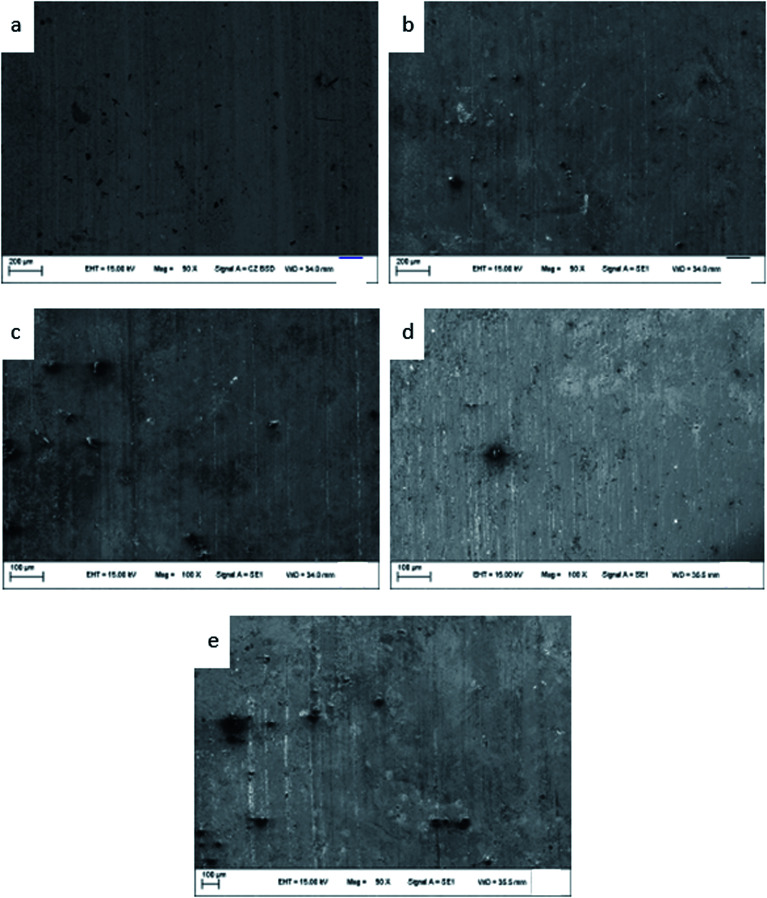
Friction topographies of ZCuPb_20_Sn_5_ with different content of P: P = 0.0 wt% (a), P = 0.05 wt% (b), P = 0.1 wt% (c), P = 0.2 wt% (d), P = 0.3 wt% (e).

The friction morphologies of the ZCuPb_20_Sn_5_ alloy with different P–Cu alloy additions are presented in Fig. 13. As it could be observed, when the P–Cu alloy was not added, friction and wear tests of the ZCuPb_20_Sn_5_ alloy were carried out. Small furrows, micro cracks and a low amount of debris were visibly adhered on the ZCuPb_20_Sn_5_ alloy friction surface. Therefore, the wear mechanism of the ZCuPb_20_Sn_5_ alloy was mainly adhesive wear. As the P content increased in the ZCuPb_20_Sn_5_ alloy, the hardness increased and a second phase (Cu_3_P) formed. Simultaneously, the wear of the ZCuPb_20_Sn_5_ alloy with the P–Cu alloy addition was more severe compared to the alloy without P–Cu alloy addition. The wear furrows became deeper on the ZCuPb_20_Sn_5_ alloy wear surface. Also, the surface layer was mostly flake-like, while the volume and quantity of the adhered particles increased. Therefore, as the P content increased, the adhesion wear of the ZCuPb_20_Sn_5_ alloy increased, along with a low abrasive wear occurrence.

During friction, the micro-bulges on the surface of the GCr15 steel ring could be pressed onto the sample layer surface under pressure, damaging the specimen surface of the contact surface, while forming furrows. During the relative sliding process, deformations, dislocations and crack nucleation were observed on the sample surface. Along with friction and wear, the dislocation amounts continuously increased and dislocation networks were formed at the grain boundary.^16^ The external continuous pressure penetrated the dislocation cell to the subsurface grain of the material. The external continuous pressure resulted in the continuous expansion of cracks, which were eventually developed into surface cracks. With repeated rolling friction, the cracks sustained a variety of complex deformation motions. Finally, the cracks eventually presented brittle fracture under external force. Certain amounts of wormlike debris were removed, while certain amounts were adhered to the grinding ring. The second phases (displaying slightly higher hardness compared to the matrix) might also act as abrasive grains that participated in the friction, which resulted in adhesive wear and a low amount of abrasive wear debris.^17^

**Fig. 14:**
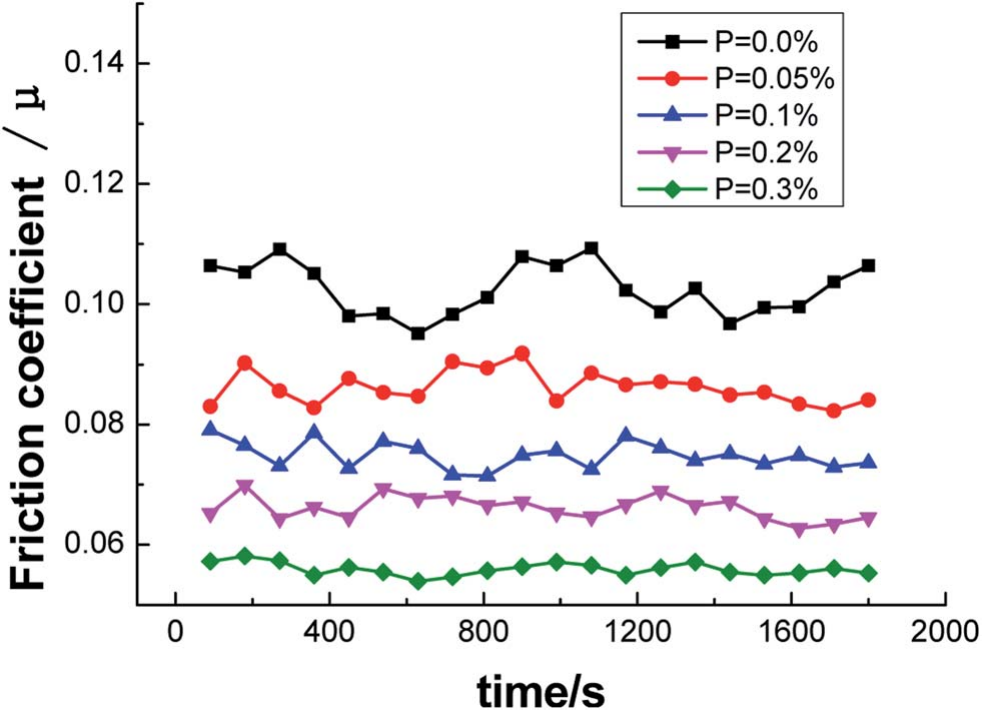
Variation curves of friction coefficient and different P content.

**Fig. 15:**
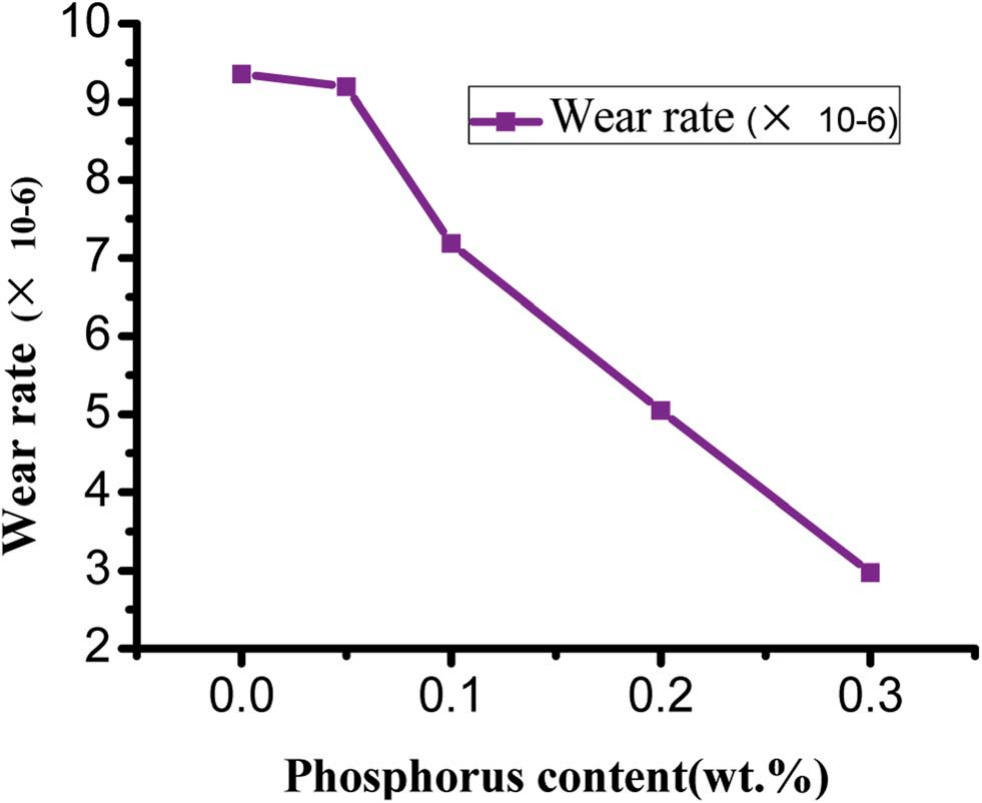
Curve of different P content and wear rate.

Friction tests of the ZCuPb_20_Sn_5_ alloy with different P contents were carried out with a friction testing machine. The test parameters were as follows: test force of 250 N, speed of 1500 rpm and duration of 30 min. The curves of friction coefficient and wear rate *versus* time are presented in Fig. 14 and 15, respectively.

From the curve of friction coefficient and P content in Fig. 14, it could be observed that the friction coefficient decreased as the P content increased. When P was not added, the friction coefficient of ZCuPb_20_Sn_5_ alloy fluctuated in the range of 0.09–0.11. The friction coefficient fluctuated in the ranges of 0.08–0.09, 0.07–0.08, 0.06–0.07 and 0.05–0.06 with the additions of 0.05%, 0.1%, 0.2% and 0.3%, respectively, while the decreased range was in-between 0.01 and stable values. It could be concluded that the friction coefficient of ZCuPb_20_Sn_5_ alloy decreased as the content increased.

Regarding the friction between the ZCuPb_20_Sn_5_ alloy and GCr15 steel ring (which had higher hardness), the friction ratio between the two contact surfaces and the positive pressure was the friction coefficient. The change in the value depended on factors, such as the sliding state, surface morphology and sliding speed of the sliding surface. During friction, the alloy surface particles were easily worn. The surface particles that were removed formed debris during friction and was adhered to the worn surface, leading the worn surface to become rough and causing fluctuations of the friction coefficient value.^18^ As the P content increased, the second phase amounts in the ZCuPb_20_Sn_5_ alloy gradually increased, whereas the grain boundary defects decreased. Therefore, during wear, the amount of surface shedding increased and the friction coefficient increased slightly when the debris participated in friction; under high-speed sliding friction, the friction coefficient increased and this was consistent with the friction morphology analysis.

From the curves of wear rate and P content in Fig. 15, it could be observed that the initial wear rate of ZCuPb_20_Sn_5_ alloy decreased slowly from 9.352 × 10^−6^ to 9.191 × 10^−6^ as the P content increased. When the P content exceeded 0.05%, the wear rate decreased abruptly as the P content increased, while the decrease rate range was approximately 2 × 10^−6^. The reason was that the P content increase led the hardness and wear resistance of P Sn bronze alloy to increase. Simultaneously, during friction and wear testing, when the lead–tin bronze material interacted with the friction pair, under the combined action of friction heat and deformation extrusion, the free lead would be gradually extruded from the alloy surface. Also, a continuous soft lead lubrication film would be formed on the friction pair surface. The soft lead lubrication film reduced the contact area among the pin and friction pair, thereby reducing the friction coefficient. Consequently, the wear rate gradually decreased as the P content increased.

A number of scholars reported that the wear rate of a metal material was inversely proportional to the hardness of the material, while it was proportional to the load. The wear rate was also related to the wear and tear mechanism of the material.^19^ As the P content increased, the second phase gradually formed at the grain boundary, reducing the bonding strength among the grains of the alloy matrix. During friction, the wear surface was easily removed and formed the debris that adhered to the worn surface. This led to adhesive wear and the wear contact area increased. On the other hand, due to heat generated by friction, the sample surface was softened, the surface strength was reduced and the adhesive metal was easily adhered to the two parts. Consequently, the direct contact among the specimen and the two components was reduced.^20^ In addition, the heat generated by friction caused the formation of an oxide layer on the alloy surface; in other words, the oxidation wear could protect the wear surface and the comprehensive effects were a reduction in the wear degree of the alloy along with a reduction in the wear rate of the alloy.

## 4 Conclusions

(1) As the P content increased, the second phases (Cu_3_P) appeared and increased in size. The microstructure of ZCuPb_20_Sn_5_ alloy was refined and the elements were evenly distributed.

(2) The strengthening effect of the second phase Cu_3_P improved the hardness of ZCuPb_20_Sn_5_. When the amount of P was 0.3 wt%, the tensile strength was 249 MPa. As the P content increased, the elongation rate increased first and consequently decreased, while the highest elongation was 17.8%.

(3) The wear mechanism of ZCuPb_20_Sn_5_ alloy was mainly adhesive wear and a small amount of debris was produced. As the the P content increased, the hardness increased and the second phases (Cu_3_P) were formed. The wear of ZCuPb_20_Sn_5_ alloy with P addition was more severe than the alloy without P addition.

(4) As the P content increased, the friction coefficient decreased. For 0.3 wt% of P, the friction coefficient was in-between 0.05 ∼ 0.06. The wear rate decreased as the P content increased. Particularly, when the P content exceeded 0.05%, the reduction range was approximately 2 × 10^−6^.

## Author contribution statement

Xin Zhang: experimental process, experimental analysis, thesis preparation; Xiao-yan Ren: experimental scheme formulation and experimental analysis; Xu Hong: experimental analysis; Xiang-yu Gao: thermodynamic calculation and experimental process.

## Conflicts of interest

The authors declare that they have no competing interests.

## Supplementary Material
